# Observations in 271 Cases of Mastoiditis

**Published:** 1908-04

**Authors:** F. P. Calhoun


### OBSERVATIONS IN 271 CASES OF MASTOIDITIS.*

F. P. CALHOUN.

Mr. President and Gentlemen:

During my interne-ship in one of the large Ear hospitals of this country, I had occasion to assist in, or operate upon 271 cases of Mastoiditis, 49 of which I operated myself, and with this ex-

*Read before Fulton County Medical Society, March 19th, 1908

perience, coupled with a number of operations I have made since my return home, I beg your attention to a few personal observations.

The cases of Mastoiditis under consideration were the acute variety, following an acute otitis, and the so-called Schwartze operation, modified or improved, was done; meaning a thorough ablation of the entire mastoid apophysis and surrounding cells.

Among the influences which played important parts in the causation of acute Mastoiditis, season was most important, for more acute ears were found during the mid-winter months, early summer, and changeable seasons, than at any other time. Age was also a factor of note, as about one-half of the total number were under ten years. The youngest patient was eight weeks, and the oldest was sixty-three years. The bacteriology of acute Mastoiditis, while a subject in itself, can be dismissed in this resume, by saying that the Streptococcus, or one of its family, and the Pneumococcus, were the exciting causes in over 95 per cent. of the cases. Experience has proven that a Streptococcus invasion is most dangerous, and it is usually quick and virulent; the Pneumococcus acts slowly as a general rule, and is not so severe, although in a few cases, acting similarly to the Streptococcus. The Streptococcus Capsulatus, an organism attracting much attention lately among Aurists, especially those bacteriologically inclined, is an organism of much treacherousness, and in northern climates acts slowly but destructively. The symptoms in these cases are mild, but its pathological scope is large. This one brief case will illustrate the working of the organism:

Three weeks before admission to the hospital, the patient, a man, complained of a fullness in his left ear, and consulted an Aurist, and the ear commenced to discharge a few days later. There was only a moderate amount of pain up to that time, when there developed mastoid symptoms. He was placed in the hospital for observation. His symptoms on admission were the usual ones for acute mastoiditis; a free muco-purulent discharge, a reddened beefy looking drum, a moderate amount of tip and antrum tenderness, and a temperature of one hundred and one. A bacteriological examination of the discharge was made after a myringotomy was done and found to be streptococcus. With five days rest in bed and appropriate treatment, all symptoms except the profuse aural discharge ceased, and the patient felt unusually well.

There was however, that same angry looking canal condition, with perhaps some oedema of the postero-superior canal wall. A second bacteriological examination was made, and the streptococcus capsulatus was found. An operation was advised solely on the bacteriological findings and the profuse, persistent aural discharge.

The operation revealed a decidedly disintegrated bone, and epidural abscess, and a large exposure of dura. The patient entirely recovered.

It might be of interest to note while discussing the bacteriology, that in all cases of erysipelas following mastoid operations (and such complications do arise in spite of all that can be done), the erysipelas occurred in patients whose aural or Mastoid pus showed streptococcus. It is my belief and contention that this is not a true erysipelas (one of contagion), but a superficial infection, and it should not excite the surgeons alarm.

I believe a bacteriological examination of the Aural and Mastoid discharge to be of an inestimable help in making a prognosis and in following the course of the disease.

It is often hard to assign true causes for Mastoiditis. Colds, infections following nasal operations, trauma, and the infectious diseases were most common, but in the vast majority of cases, no cause could be determined.

Patients sought medical advice, either on account of pain in the ear, in the region of the mastoid or its vicinity, aural discharge, fever or swelling. Any one or combination of these symptoms were present, or all combined. Generally, adults came on account of pain, and babies were brought on account of post-aural swelling. Chills, fever, sweats, nausea, vomiting and vertigo were not uncommon symptoms in uncomplicated Mastoiditis, and optic neuritis has been observed in small extradural abscesses.

Temperature was seldom normal, very rarely sub-normal, but usually between 99 and 101. Where a temperature was 103 or above, suspicion was at once aroused, fearing some complication. There were five cases which, on admission to the hospital, showed a temperature between 103 and 105, and four had Sinus Thrombosis, and one Meningitis and Brain Abscess. Swelling was present over or about the Mastoid in about 50 per cent. of the cases, and pus was found 88 times in 133 cases having swelling, and 54 of the 88 cases with pus were children under five years. It is thus seen that one-half of the Subperiosteal abscess formation occurred in babies. Swelling was a symptom of Matoiditis in infants and children, and was present in about 90 per cent. Swelling usually occurred as an early symptom in children and infants, as did the abscess formation, and a late manifestation in adults.

Palpation was the most valuable sign of Mastoid involvement, and even then in a few cases it was misleading entirely. The tenderest portion did not always indicate the location of the greatest destruction. The tip was a maximum point of sensitiveness in relation to the antrum an post tip in the relative proportion of 3,2, and 1. Post tip tenderness usually indicates cases of long standing or great destruction of the mastoid.

Frequently cases were found in which there was no tenderness of the mastoid on palpation, yet an operation was indicated on account of the profuse continuous discharge, septic temperature or headache. Nervous hysterical women misled two good aurists in two instances, and perfect normal mastoids were opened.

Sagging of the postero-superior canal wall, considered by many a positive sign of Mastoiditis, and an operative symptom, occurred in 40 per cent. of the cases examined, although the majority of these cases were in infants with a sub-periosteal abscess.

The aural examination showed the usual appearances of an acute purulent ear: Red, bulging drum, with or without perforation, and the land marks generally destroyed. The discharge ranged from a scanty serous nature in early cases, to a thick, yellow purulent one in cases of long standing.

Facial paralysis occurred as a symptom in four cases; they were in cases of long duration, and this symptom was a late one. All symptomatic paralyses recovered after operation.

The question naturally arises what constitutes operative symptoms of Mastoiditis in the absence of cardinal signs. With headache, temperature, profuse aural discharge, mastoid swelling, and pain on palpation, there can be little doubt as to what should be done. Many cases have but little temperature, although considerable discharge and much tenderness; such a case of long standing would be considered an operative one. Again, there is no symptom but a profuse aural discharge out of proportion to the usual tympanic infection. Such a quantity of pus could only come from a disintegrating mastoid, and with a microscopical examination showing Streptococcus Capsulatus in a case of six weeks' duration, I would operate.

Mastoid tenderness, while the most valuable of all symptoms, can not be absolutely relied upon, for in both cases of hysteria mentioned, this was the leading symptom.

Blood counts, with an estimate of the percentage chiefly of the Polynuclearleucocytes is at times misleading, and it, too, does not always give positive evidence. However, in an important case with doubtful operative Mastoidital symptoms with one or two blood examinations, showing a Leucocytosis and a Polynuclear account of 79 per cent., or over, I would be inclined to operate.

In two cases of doubtful operative Mastoiditis, one of a Streptococcus, the other of a Pneumococcus infection, I would more likely wait 24 to 48 hours on the Pneumococcus than upon its more virulent kin.

In addition to what has already been said relative to signs and symptoms, the patient's general condition, his facial expression, has much to do with whether one should operate now or await events for a few days.

To my mind, it is not the question of how little or how much pus one will find in the Mastoid, but will the patient recover without operative interference; and in closing this part of the paper, I would say that no patient should be considered an operative one until a free Myringotomy had been made, and that is most difficult to do without general anæsthetic.

The Mastoid operation of to-day (the improved Schwartze) is so different from the work of surgeons of ten to fifteen years ago, that it is not surprising that a number of cases did not recover, that cases were left with posterior discharging sinuses or chronic discharging ears. As it is, in this series of 271 cases, as reviewed by the writer, 73.30 per cent. healed, 10.30 per cent. were in a state of healing when last seen, 8.40 per cent. were unhealed and no evidence of a permanent cure, while 5 per cent. had healed, but their ears still discharging. 50 per cent. of the cases unhealed occured in children under five years of age, due largely to carelessness and neglect by their parents after dismissal from the hospital.

This improved Schwartze operation, taking the place of the Wilde's incision and the mere curettment of the Antrum, had reduced the percentage of failures in one series that I had occasion to follow from 25 per cent. to 13 per cent. This could even be less if one's private statistics could be consulted.

This improved Schwartze operation, consisting in the total ablation of all diseased structure, including the removal of the tip, is almost universally practised, and credit is mostly due our American operators.

I have had occasion to follow many cases of operative Mastoiditis where the work was done in both extremes; from the most conservative, almost bordering upon a mere removal of a portion of the cortex, and a slight curettment of the antrum, to the very extreme—a total ablation. With but one or two exceptions, I have yet to see a recurrence or a failure in healing after a thorough operation.

The operation itself can be accomplished in any number of ways, best I think with these few instruments, viz.: the large gouge, to establish the initial groove; the Mathieu Rongeur, to remove the cortex, and the back bent round curette of Richards, to remove the soft cellular structure. In removing the tip, the muscular attachment of the Sterno-Mastoid should not be severed, or cut, but gently detached with its periosteal attachment. Less pain in the neck following operation, and less danger with secondary wound infections are the reasons.

Best results are obtained where the wound is packed with iodoform gauze, dressed on the fourth day, and redressed every other following day, unless otherwise indicated. Where dressings are left longer, granulations grow into the meshes of the gauze, giving the patient much pain and discomfort at the dressings. If the aural secretion does not subside in a reasonable length of time, the bulky ear dressing should be removed in part and the canal exposed and irrigated many times a day, otherwise, it might be a means of promoting a secondary infection, leaving a posterior discharging sinus. From eight to ten weeks is an average time for an uncomplicated Mastoid wound to heal.

There are three post operative complications, that at times prove quite troublesome, viz.: Facial paralysis, treated by the galvanic or faradic current, and massage; impaired hearing and tinnitus occurring in 6.4 per cent. and 5 per cent. of this series respectively, treated by tympanic inflations and vaporization; and impacted cerumen, in a distorted or drooping canal wall in 13 per cent. This complication, while not serious, is an annoyance, and is most likely due to the traumatism to the canal wall during the operation, disturbing the normal outlets to the deep ceruminous glands.

